# Serum TNF-Alpha Level Predicts Nonproliferative Diabetic Retinopathy in Children

**DOI:** 10.1155/2007/92196

**Published:** 2007-05-17

**Authors:** Katarzyna Zorena, Jolanta Myśliwska, Małgorzata Myśliwiec, Anna Balcerska, Łukasz Hak, Paweł Lipowski, Krystyna Raczyńska

**Affiliations:** ^1^Department of Immunology, Medical University of Gdańsk, Dębinki 1, 80-210 Gdańsk, Poland; ^2^Diabetological Department, Clinic of Pediatrics, Hematology, Oncology and Endocrinology, Medical University of Gdańsk, 80-210 Gdańsk, Poland; ^3^Department and Clinic of Ophthalmology, Medical University of Gdańsk, 80-210 Gdańsk, Poland

## Abstract

The aim of this study was identification of the immunologic markers of the damage to the eye apparatus at early stages of diabetes mellitus (DM) type 1 children. One hundred and eleven children with DM type 1 were divided into two groups: those with nonproliferative diabetic retinopathy (NPDR) and without retinopathy. All the children had their daily urine albumin excretion, HbA1c, C-peptide measured, 24-hour blood pressure monitoring, and ophthalmologic examination. Levels of TNF-*α*, IL-6, and IL-12 in serum were measured by ELISA tests (Quantikine High Sensitivity Human by R&D Systems, Minneapolis, Minn, USA). The NPDR children demonstrated a significantly longer duration of the disease in addition to higher HbA1c, albumin excretion rate, C-reactive protein, systolic blood pressure, as well as TNF-*α* and IL-6 levels than those without retinopathy. The logistic regression revealed that the risk of NPDR was strongly dependent on TNF-*α* [(OR 4.01; 95%CI 2.01−7.96)]. TNF-*α* appears to be the most significant predictor among the analyzed parameters of damage to the eye apparatus. The early introduction of the TNF-*α* antagonists to the treatment of young patients with DM type 1 who show high serum activity of the TNF-*α* may prevent them from development of diabetic retinopathy.

## 1. INTRODUCTION

Diabetic retinopathy usually does not occur during the first five years from the diagnosis of diabetes mellitus (DM) type 1, whilst after 10 years of diabetes it is present in over 50% of patients, being the most common cause of loss of vision [1]. Regardless numerous studies carried out worldwide, daily urine albumin secretion remains to be standard marker of nephropathy development, while ophthalmologic examination stands for diagnosis of retinopathy [2, 3]. Presently, there is no satisfactory pharmacologic therapy and laser therapy remains to be the leading treatment option in management of DM1 retinopathy in children. Yet still the therapy is aimed at limiting the damage and not at preventing it [3, 4]. Early detection of the individual tendency for development of diabetic retinopathy, especially before evident changes become visible at the fundus of the eye, is extremely significant in children and adolescent with DM type 1. Most of the published works focus on the role of cytokines in the development of proliferative retinopathy in adults, thus such analysis is limited to the late stages of the retinopathy [5–8]. It has been suggested that hyperglycemia may lead to the activation of proinflammatory cytokines that are crucial for development and progression of retinopathy [9, 10]. One of the glucose toxic mechanisms, the protein glycation, is associated with cytokines: tumor necrosis factor-alpha (TNF-*α*), interleukin-6 (IL-6), and interleukin-12 (IL-12) that are substantial factors in the development of diabetic microangiopathy [9–11]. TNF-*α* and IL-6 may act as local intensification signals in pathological processes associated with chronic eye inflammation [5, 6]. It has been shown in NOD-mice that also IL-12 is associated with rapid diabetes development [12]. Therefore, the purpose of this work was the identification of the early immunologic markers of the damage to the eye apparatus in diabetic children.

## 2. PATIENTS AND METHODS

One hundred and eleven children (age 15 ± 2 years) with DM type 1 were recruited from The Diabetological Department, Clinic of Pediatrics, Hematology, Oncology and Endocrinology Medical University of Gdańsk. Type 1 DM, was defined in accordance with the criteria of the American Diabetes Association [13, 14].

## 3. LABORATORY EXAMINATIONS

HbA1c was measured by an immunoturbidometric method using Unimate 3 set (Hoffmann-La Roche AG, Germany) with a normal range of values 3.0–6.0%. Fasting glucose was measured by enzymatic test (Roche Diagnostics GmbH, Germany). Level of CRP was measured with immunochemical system (Beckman Instr. Inc., Ireland). Level of C-peptide was below 0.5ng/ml in all children with type 1 DM. The systolic and diastolic blood pressures were measured using automatic 24-hour ambulatory blood pressure monitoring (ABPM) and all the average values of the blood pressure were expressed in the centile charts. The urinary albumin excretion was expressed as the average of the three 24-hour collections obtained during 6 months prior to the enrolment into the study. Classified as microalbuminuria were the cases when at least in two out of three urine samples albumin excretion was between 30–299 mg/24 hours. The urinary albumin excretion is measured by immunoturbidometric assay using Tina-quant (Boehringer Mannheim GmbH, Germany). Serum level of creatinine was measured using CREA assay system (Boehringer Mannheim GmbH, Germany). All patients with type 1 DM required insulin treatment (0.87 ± 0.24 U/kg of the body weight).

## 4. OPHTHALMOLOGIC EXAMINATION

In all children with type 1 DM the ophthalmologic investigation was performed. This included visual acuity tests, intraocular pressure, and anterior segment estimation done by slit lamp (TOPCON SL-82, Japan). The fundus examination was done after installation of 1% Tropicamid to obtain sufficient mydriasis. The examination was performed by using the +90D lens (Ocular Instruments Inc., Bellevue, Wash, USA). Digital camera (Topcon IMAGEnet 2000 Japan) was used to perform the fluorescein angiography.

The analysis of the eye fundus pictures was based on The International Diabetic Retinopathy Division: 1) nonproliferative retinopathy with or without maculopathy, 2) preproliferative retinopathy, 3) proliferative retinopathy [15]. According to the ophthalmological examination, the diabetic children were divided into 2 groups: with diabetic retinopathy (21 children) and without retinopathy (90 children). In 21 cases, typical symptoms of nonproliferative diabetic retinopathy were proven. Additionally, 16 children of the group with nonproliferative diabetic retinopathy had microalbuminuria and 7 children were presented with arterial hypertension, while none of the children without retinopathy presented clinical signs of diabetic nephropathy such as microalbuminuria or arterial hypertension.

A group of 41 healthy children (age 14 ± 2 years) volunteered as the control group. They were apparently healthy as based on their medical examination. Written, informed consent was obtained from all children (or from their parents) participating in the study. This study was approved by The Ethics Committee of The Medical University of Gdańsk NKEBN/610/2003/2004 and the investigation was carried out in accordance with the principles of the Declaration of Helsinki as revised in 1996.

## 5. SAMPLE COLLECTION

Plasma samples were collected from the 152 children. Blood samples were immediately placed on ice, clarified by centrifugation at 3.000 × *g* for 5 minutes at 4°C, and kept frozen at −80°C until assay.

## 6. DETERMINATION OF TNF-*α*, IL-6, IL-12 LEVELS

Serum levels of cytokines TNF-*α* IL-6, IL-12 were measured by immunoenzymatic ELISA method (Quantikine High Sensitivity Human by R&D Systems, Minneapolis, Minn., USA) according to manufacturer protocol. Minimum detectable concentrations were determined by the manufacturer as 0.12 pg/ml, 0.03 pg/ml, and 1.0 pg/ml, respectively. Intra-assay (2.6 for TNF-*α*; 1.6 for IL-6; 1.1 for IL-12) and interassay (7.4 for TNF-*α*; 6.4 for IL-6; 7.1 for IL-12) precision performances of the assays were determined on 20 replicates from the quality control data of the laboratory.

## 7. STATISTICAL ANALYSIS

The results were analyzed using The Statistical Version 7.0 program (StatSoft Polska ). The Shapiro-Wilk test was used to evaluate normality of variables. The differences between the groups were calculated with Student *t* or the nonparametric *U*-Mann-Whitney tests.

A logistic forward regression analysis was used to assess the association between all clinical and inflammatory parameters and retinopathy with a *P* < .05 for entry. Risk for retinopathy was estimated by odds ratios (ORs) with 95% confidence intervals (CIs).

The χ^2^ person test was applied for calculating differences between numbers of children with and without detectable cytokines in serum. The results of Student *t* test were presented as arithmetic means ±SD. The results of the *U*-Mann-Whitney test were presented as median (min-max). In all analyses a two-tailed significance level <.05 was regarded as statistically significant.

## 8. RESULTS

The NPDR children demonstrated a significantly longer duration of the disease in addition to higher HbA1c, albumin excretion rate, C-reactive protein, as well as systolic blood pressure than those without retinopathy. There were no significant differences between both groups in the serum creatinine and diastolic blood pressure. Moreover, the NPDR children demonstrated a significantly higher HbA1c, C reactive protein, albumin excretion rate, creatinine in serum, systolic and diastolic blood pressures as compared to the healthy group (Table 1).

The serum levels of TNF-*α* and IL-6 in the group with NPDR were significantly higher as compared with children without retinopathy. On the other hand, the IL-12 levels were higher in the NPDR group with regard to the patients without retinopathy, however the difference was statistically insignificant. Children without retinopathy were characterized by significantly higher TNF-*α*, IL-6, as well as IL-12 serum levels in relation to the healthy controls (Table 2).

As many as 76% of the NPDR children had a detectable serum level of TNF-*α* while in the group without retinopathy only 34% were TNF-*α* positive. This stands in clear contrast to the healthy group which was absolutely (100%) negative with respect to serum TNF-*α*. The differences between children with and without NPDR were statistically significant. However, all the NPDR children (100%) had a detectable serum level of IL-6 and 70% of children without retinopathy were IL-6 positive. The differences between both diabetic groups were statistically significant. In the healthy group only 2.85% were IL-6 positive. There were no differences between percentages of the IL-12 positive children in the groups with NPDR and without retinopathy, 43% and 24%, respectively.

Results of the factors predisposing to diabetic retinopathy development in DM1 children are summarized in Table 3.

## 9. DISCUSSION

We have examined a group of 111 children diagnosed with DM type 1, including 21 (26%) children with recognised nonproliferative diabetic retinopathy. In relation to those without retinopathy, the NPDR children demonstrated significantly longer duration of the disease, they had higher HbA1c, urine albumin excretion rate, CRP level, as well as the systolic blood pressure. TNF-*α* was detectable in 76% of the NPDR children and only in 34% of the DM type 1 children without retinopathy. Statistical analysis disclosed TNF-*α* as the only independent early marker indicating occurrence of changes at the fundus of the eye.

In a few studies published in recent years authors reported detectable TNF-*α* in the serum of children with long-standing diabetes, however these patients had no concomitant diabetic complications [16–19]. The Chinese scientists analyzed a group of children of less than 5 years of diabetes mellitus type 1 duration having no late complications of the disease [17]. They showed that TNF-*α* serum levels did not differ from those of the healthy children. The Italian group reported results of a study in which TNF-*α* and IL-6 serum levels were compared between the groups of children being less than one year or over five years from the DM type 1 diagnosis and healthy controls. Children being less than one year form the disease diagnosis presented higher TNF-*α* serum levels in comparison to the other two groups [18]. On the other hand a different group reported that TNF-*α* serum level was found lower than in the healthy controls [19].

The mechanism of TNF-*α* contribution to diabetic retinopathy is not fully elucidated. It has been suggested that hyperglycaemia may lead to the activation of proinflammatory cytokines that are crucial for micro- and macroangiopathy developments [9, 10]. In diabetic patients an increased synthesis of the macrophage's RAGE receptors, which bind final glycation products, has been noted [10]. The RAGE receptors signalize the proinflammatory cytokines' cascade induction, including TNF-*α*, IL-6, and IL-12 [9–11]. These cytokines may mediate the synthesis of acute phase proteins which are able to initiate and support inflammatory process in the vascular wall. As a consequence, an increased expression of intercellular adhesion molecule-1 (ICAM-1) and vascular adhesion molecule-1 (VCAM-1) on endothelial cells is induced, which serves as chemoattractants for monocytes and other inflammatory cells [1, 11, 20].

In our study serum IL-6 level was more elevated in NDPR group than without retinopathy children and similar relations concerned the IL-6 positive sera in these groups. On the other hand, IL-6 did not reach a status of an independent retinopathy risk factor. The negative role of IL-6 is also associated with its effect on endothelial dysfunction with an increased permeability and enhanced expression of adhesion molecules on these cells [18, 21, 22]. Elevated levels of IL-6 both in serum and in vitreous fluid have been detected in proliferative retinopathy in adult patients [23, 24]. However, there was no report published in the available literature on the role of IL-6 at the early stages of the diabetic retinopathy development in DM type 1 children. The NPDR children, apart from elevated TNF-*α* and IL-6 levels, did not differ in the IL-12 concentration. Formerly, the role of IL-12 as a proinflammatory cytokine in the insulin-dependent diabetes pathogenesis was shown using the NOD model [12] and in some limited studies in humans [25, 26]. Winkler et al. showed that IL-12 may also contribute to the pathogenesis of diabetic retinopathy more likely as a part of the consecutive immunological response mechanisms rather than a predisposing factor [26].

On the account of the presented data it appears that, among the analyzed parameters, TNF-*α* is the most significant predictor of damage to the eye apparatus. The early introduction of the TNF-*α* antagonists to the treatment of young patients with DM type 1 who show high serum activity of the cytokine may prevent development of diabetic retinopathy.

## Figures and Tables

**Table 1:** Clinical parameters of the DM type 1 children and healthy group. Values are presented as means ± SD.

Clinical parameters	Children with NPDR	Children without retinopathy	Healthy group	Statistical significance

Number of children	21	90	41	
Age (years)	15 ± 2	14 ± 3	14 ± 2	NS
				NS
Duration of DM (years)	9 ± 4	5 ± 3	—	*P* < .001*
				*P* < .001**
HbA1c (%)	9.7 ± 1.8	8.2 ± 1.7	4.2 ± 0.3	*P* < .001*
				*P* < .001**
C-reactive protein (mg/L)	2.3 ± 1.0	1.4 ± 0.8	0.3 ± 0.04	*P* < .001*
				*P* < .001**
Albumin excretion rate (mg/24)	34 ± 21	18 ± 12	3 ± 1	*P* < .001*
				*P* < .001**
Creatinine in serum (*μ*mol/L)	0.9 ± 0.9	0.9 ± 0.8	0.5 ± 0.1	NS
				*P* < .001**
Systolic blood pressure (mm/Hg)	122 ± 10	155 ± 11	110 ± 10	*P* < .001*
				*P* < .001**
Diastolic blood pressure (mm/Hg)	73 ± 10	70 ± 9	68 ± 8	NS
				*P* < .001**

*Differences between children with nonproliferative retinopathy and without retinopathy.

**Differences between children with nonproliferative retinopathy and healthy group.

**Table 2:** Circulating levels of cytokines in serum of DM type 1 children and healthy group. Values are presented as means ± SD.

Level cytokines	Children with NPDR	Children without retinopathy	Healthy group	Statistical significance

N	21	90	41	
TNF-*α* (pg/mL)	1.7 ± 1.4	0.6 ± 0.8	0.0	*P* < .001*
				*P* < .001**
IL-6 (pg/mL)	3.9 ± 1.2	1.8 ± 0.9	0.5 ± 0	*P* < .001*
				*P* < .001**
IL-12 (pg/mL)	1.8 ± 1.6	1.2 ± 1.5	0.0	NS
				*P* < .001**

*Differences between children with nonproliferative retinopathy and without retinopathy.

**Differences between children with nonproliferative retinopathy and healthy group.

**Table 3:** Unadjusted retinopathy risk gradation.

Parameters	OR	95% Cl	Statistical significance

Serum level of TNF-*α* (pg/mL)	4.01	2.01–7.96	.0001
C-reactive protein (mg/L)	2.55	1.54–4.2	.0001
HbA1c (%)	1.58	1.22–2.06	.0004
Serum level of IL-6 (pg/mL)	1.52	1.11–2.07	.007
Duration of DM (years)	1.39	1.39–1.18	.005
Age (years)	1.25	1.07–1.46	.003
Systolic blood pressure (mm/Hg)	1.08	1.02–1.13	.03
Albumin excretion rate (mg/24)	1.01	1.00–1.03	.03
Diastolic blood pressure (mm/Hg)	1.03	0.98–1.08	.2
			NS
Creatinine in serum (*μ*mol/L)	1.0	0.9–1.0	.3
			NS
Serum level of IL-12 (pg/mL)	0.9	0.6–1.2	.7
			NS

## ABBREVIATIONS

DM: Diabetes mellitus
NPDR: Nonproliferative diabetic retinopathy
TNF-*α*: Tumor necrosis factor-alpha
IL-6: Interleukin-6
IL: Interleukin-12
ABPM: Ambulatory blood pressure monitoring
RAGE: Receptor for advanced glycation end product
ICAM-1: Intercellular adhesion molecule-1
VCAM-1: Vascular adhesion molecule-1
